# Quantitative Evaluation of Irradiated Ductility Degradation Using the Indentation Technique Combined with Numerical Experiments

**DOI:** 10.3390/ma17235925

**Published:** 2024-12-03

**Authors:** Takashi Wakui, Shigeru Saito, Masatoshi Futakawa

**Affiliations:** J-PARC Center, Japan Atomic Energy Agency, Ibaraki 319-1195, Japan; saito.shigeru@jaea.go.jp (S.S.); futakawa.masatoshi@jaea.go.jp (M.F.)

**Keywords:** ductile property, indentation test, tensile test, numerical experiment, inverse analysis, ductile failure criterion

## Abstract

The ductile properties of irradiated materials are among of the important indicators related to their structural integrity. These properties are generally determined by performing tensile tests on irradiated materials in the irradiation environment. Indentation tests are used for evaluating ductile properties easily and rapidly. Constants in the Swift-type material constitutive equation were identified via inverse analysis using the Kalman filter, such that the numerical experimental results reproduced the indentation test results. Numerical tensile experiments were conducted using stress–strain curves with the identified constants to obtain nominal stress and strain curves. The identified yield stress, work hardening coefficient, and exponent were 200–1000 MPa, 1100–1500 Ma, and 0.5–0.7, respectively. Furthermore, two methods were proposed for evaluating the total elongation. Method I was used to calculate the total elongation based on the relationship between the total and uniform elongations obtained from the tensile tests performed on irradiated materials. Method II was used to determine the total elongation from the ductile failure criterion based on the relationship between the stress and strain states in the tensile specimen model using the numerical tensile experiment and failure strain evaluated from actual tensile experiments. Evaluated minimum total elongation was 10%. The evaluation results for ion-irradiated materials were similar to the tensile test results for irradiated materials.

## 1. Introduction

Ductility is crucial in structural materials because it relieves stress concentrations. The ductility of metals has been categorized, based on their total elongation to failure, as brittle (<1%), limited ductility (1–10%), and adequate ductility (>10%) [1]. Localized yielding in metals with adequate ductility reduces the severity of design discontinuities. The total elongation of materials decreases in irradiation environments such as fast breeder reactors. In fast breeder reactors, materials are required to have 10% total elongation to maintain adequate ductility during operations [2]. The dose limit of fast neutrons that can ensure this standard value was determined based on its influence on the degradation in elongation. Tensile testing is used for determining the ductility of small material specimens. Indentation tests are performed to evaluate the mechanical properties of thin films and microscopic materials. Indentation tests are relatively complex and time-consuming compared with tensile testing on small specimens. This test does not require specially shaped test specimens or a fairly well-specified boundary condition between the specimens and loading jigs. The indent size can be measured using the Vickers and Brinell hardness tests to determine the hardness from its surface area. The hardness value can be then used to calculate the approximate proof stress and ultimate strength using an empirical formula [3]. Load and depth are continuously measured using the Vickers or Berkovich indenter, and indentation hardness and modulus are evaluated from the unloading curve [4]. By converting the projected contact area used for calculating the indentation hardness into surface area, the value equivalent to Vickers hardness can be calculated. The relationships between the stress–strain curves of materials and load–depth curves measured during indentation tests have also been previously investigated. Moreover, various methods for determining the stress–strain curve from the load–depth curve have been examined since the end of the 20th century [5]. In a numerical experiment using a sharp indenter such as the Vickers indenter, the load–depth curves evaluated based on multiple stress–strain curves yielded almost the same results. These findings indicated that obtaining a stress–strain curve from the load–depth curve is difficult [5]. As the stress and strain fields beneath a sharp indenter are scale-independent, increasing the indentation depth only slightly impacts mechanical characterization. For example, the stress and strain fields created by indenting twice as deep will only be twice as extensive, and the loads will only be four times larger. In contrast, as the stress and strain fields beneath the indenter depend on the apex angle of the indenter, methods were proposed for evaluation of the constants in material constitutive equations using sharp Berkovich indenters [6,7,8] and conical indenters [9] with different apex angles or a sharp indenter with two apex angles [10]. Furthermore, evaluations have also been conducted using combinations of a conical indenter and a Berkovich indenter [11,12], a conical indenter and a flat indenter [13], and a spherical indenter and a flat indenter [14]. Furthermore, a spherical indenter has an infinite number of contact angles, and the stress and strain fields beneath the spherical indenter change qualitatively as the indentation depth increases. As such, evaluation methods using a spherical indenter have been suggested.

The evaluation methods can be categorized into two categories. Category 1 involves evaluating the stress–strain relationship using an analytical formula based on the in-formation obtained from each unloading curve in a cyclic indentation test, in which the indentation load is increased stepwise [15,16]. Numerical experiments using the finite element method [17] and indentation tests with different indenter diameters [18] were conducted to verify the validity of the evaluation method. Additionally, indentation tests combined with microstructural observations were conducted to investigate the relationship with the metal structure [19]. Plastic strain is calculated using Tabor’s empirical equation, wherein the ratio of the diameter of the spherical indenter and the residual contact diameter are evaluated during unloading from the cyclic load–depth curve [20]. Plastic stress is assumed to be proportional to the mean nominal pressure calculated by dividing the load by the residual contact area. This evaluation method is ideal because stress–strain curves can be rapidly derived using well-defined analytical formulations. However, the stress and strain fields beneath the spherical indenter change in a complex manner with the indentation depth; therefore, evaluating the realistic stress–strain curves is difficult, although complex analytical formulations are used.

Category 2 involves evaluating stress–strain curves via inverse analyses using the finite element method, which can reproduce evolving stress and strain fields beneath spherical indenters [21,22,23,24,25,26]. This method is simple and yields clear results. However, performing inverse analysis for deriving optimum stress–strain curves from measured load–depth curves is a major challenge and has ambiguities such as the fact that different stress–strain curves yield essentially the same load–depth curves. These evaluation methods have been studied and applied to both metallic and brittle materials [27,28]. The Kalman filter and the least squares method are used for deriving optimal stress–strain curves via inverse analysis [21,29]. Neural networks and machine learning have also been used recently to minimize the difference between load and depth curves obtained via experiments and the finite element method [30,31,32,33,34]. In these evaluation methods, the tensile properties of materials up to uniform elongation before necking are evaluated. However, the total elongation to failure, which is an important ductile property, has not been evaluated.

Tensile tests on small specimens irradiated under conditions simulating the actual irradiation environment have been performed to investigate their degradation due to irradiation [35,36,37,38,39,40]. Material degradation due to radiation environment has been simulated via ion irradiation because the dose of ion-irradiated specimens is low, and these specimens are easy to handle. As the irradiated volume is limited to the surface layer of the test specimen, and tensile tests are difficult to perform, indentation tests were performed instead [41,42]. Microindentation tests are ideal for evaluating the mechanical properties of microscopic materials; moreover, material hardness is affected by the material properties altered due to irradiation [42].

In this study, we determined constants in the material constitutive equation by combining the indentation technique with inverse analysis using the Kalman filter; moreover, numerical tensile experiments were conducted on ion-irradiated materials with irradiation damage in the thin damage region. Then, their mechanical properties were assessed via numerical tensile experiments using the material constitutive equation with identified constants. These findings were compared with previously reported results of tensile tests performed on irradiated materials. Furthermore, two methods for evaluating the total elongation were proposed as follows: Method I is based on the relationship between total and uniform elongations, and Method II uses the ductile failure criterion based on the relationship between the stress state in the tensile specimen and failure strain.

## 2. Evaluation Methods

### 2.1. Flow of Ductile Properties Evaluation

**Figure 1:**
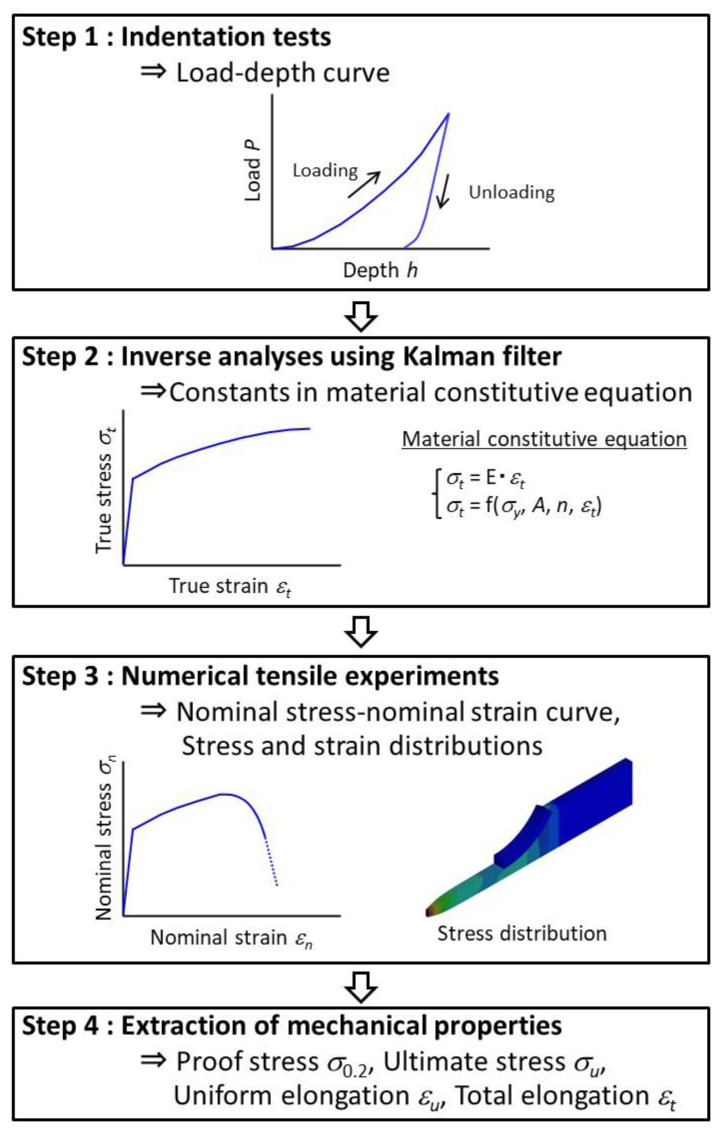
Process flow of mechanical property evaluation.

The flow of evaluating mechanical properties is shown in Figure 1. It comprises four steps as follows:

(1) Step 1: Indentation tests
  Indentation tests were performed using spherical and Berkovich indenters to obtain load and depth (*L*–*D*) curves. Elastic modulus was evaluated from the unloading part in the *L*–*D* curves measured using the Berkovich indenter [4].
(2) Step 2: Inverse analyses using the Kalman filter
  Inverse analyses using the Kalman filter [43] were conducted to identify constants in the material constitutive equation representing the relationship between true stress *σ_t_* and true strain *ε_t_*. Herein, Swift-type constitutive equations (Equations (1) and (2)) [44] were used.
  (1) $$\sigma_{t}=A{\left(\varepsilon_{0}+\varepsilon_{t}\right)}^{n},$$
  (2) $$\varepsilon_{0}={\left(\sigma_{y}/A\right)}^{1/n},$$
  where *A*, *n*, and *σ_y_* are the work hardening coefficient, work hardening exponent, and yield stress, respectively.
(3) Step 3: Numerical tensile experiments
  Numerical experiments simulating the tensile tests were conducted based on the constants identified in Step 2 to obtain nominal stress–nominal strain curves as well as stress and strain distributions at the center of tensile test specimens.
(4) Step 4: Extraction of mechanical properties
  Proof stress *σ*_0.2_, ultimate strength *σ_u_*, and uniform elongation *ε_u_* were derived from the nominal stress and nominal strain curves obtained in Step 3. Furthermore, total elongation *ε_t_* was estimated using the two methods described in Section 2.2. Uniform and total elongations are defined as the nominal strains at ultimate strength and at failure in this study.

### 2.2. Evaluation Method of Total Elongation

**Figure 2:**
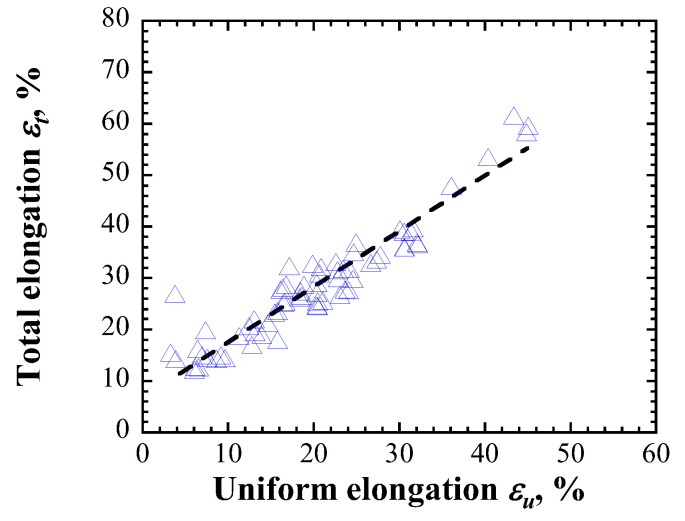
Relationship between total and uniform elongations.

Two methods were proposed herein to determine total elongation. Method I uses an empirical equation based on the relationship between total and uniform elongations. Figure 2 shows the tensile test results of specimens cut from used mercury targets for a spallation neutron source [45,46]. The total and uniform elongations exhibit an approximately linear relationship (Figure 2). The regression line shown in Equation (1) was obtained as follows:

(3) $$\varepsilon_{t}=1.08\varepsilon_{u}+6.67,$$

therefore, total elongation can be calculated from the derived uniform elongation using Equation (3).

**Figure 3:**
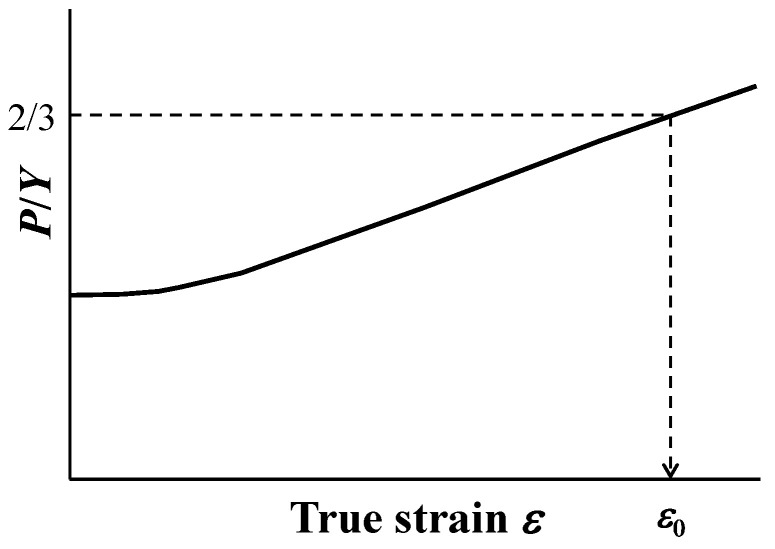
Relationship between stress triaxiality and true strain at the center of the tensile test specimen.

Method II employs a homologous ductile fracture criterion [47] based on tensile test results and numerical experiments for four ductile materials [48,49]. From the numerical analysis, the mean stress *P*, effective stress *Y*, and true strain *ε* at the center of the tensile specimen are calculated, and the relationship between *P*/*Y* and true strain is shown in Figure 3. *P*/*Y*, defined as the ratio of mean stress to effective stress, refers to stress triaxiality. The relationship between the fracture strain *ε_f_* evaluated from the tensile test results and the strain *ε*_0_ at *P*/*Y* = 2/3 (i.e., pure biaxial stress state) of the tensile specimen using the numerical experiments can be summarized as follows (Equation (4)).

(4) $$\varepsilon_{0}=0.109\varepsilon_{f}+0.731{\varepsilon_{f}}^{2}+0.284{\varepsilon_{f}}^{3}$$

it was assumed that failure occurred when the strain at the center of the tensile test specimen reached the failure strain, and the total elongation was evaluated based on that elongation.

## 3. Application of Evaluation Technique on Ion-Irradiated Materials

### 3.1. Materials and Experiments

The material evaluated in this study for the mercury target vessel is 316L austenitic stainless steel; it is used for evaluating the vessel’s lifespan. The size of each small plate specimen is 3 mm × 6 mm × 0.5 mm. The surface to be irradiated with ions was subjected to mechanical polishing, followed by electric polishing. Ion irradiation with an ion beam of 12.0 MeV nickel and 1.0 MeV helium at a temperature of 673 K was conducted at Takasaki Ion Accelerators for Advanced Radiation Application, Japan [41]. The irradiated area was the central portion (2 mm × 0.5 mm) of the polished surface (6 mm × 0.5 mm). The depth profiles of atomic displacement damage by nickel ions and helium ion concentration were determined using the TRIM code [50]. In the irradiated area with a depth of ~2.6 μm, the displacement damage intensity increased with increasing depth; it reached its maximum value at a depth of ~2 μm. Two irradiated specimens were prepared with maximum displacement damage intensities of 5 and 35 dpa. The maximum injection rate of the helium ions was 50 appm/dpa.

Indentation tests were conducted 10 times at intervals of 50 μm in the unirradiated and irradiated areas of the specimen surface using a dynamic ultra-microhardness tester (Shimadzu, DUH-211S, Shimadzu Corporation, Kyoto, Japan [51]). A conical indenter with a tip radius of ~5 μm was used. The maximum indentation load was set to 14.7 mN to ensure an indentation depth of at least 0.5 μm. Due to this indentation depth, the plastic region under the indenter reached approximately twice the ion irradiation depth of ~2.6 μm.

### 3.2. Numerical Experiments

Numerical experiments were conducted using an explicit EFM code, LS-DYNA_971 (Livemore Software Technology Corporation, Livemore, CA, USA) [52], which enabled the robust analysis of large deformation accompanying with the contacting behavior.

**Figure 4:**
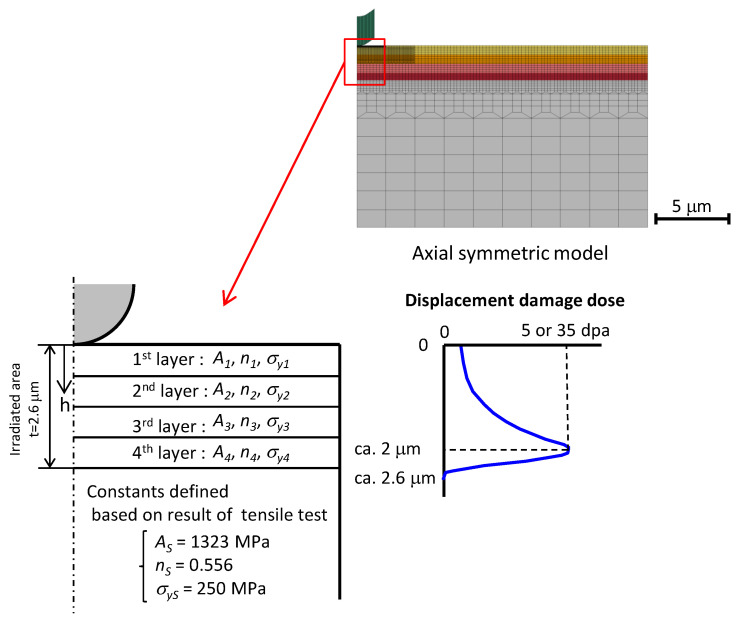
Numerical experimental model simulating indentation tests. The colors of the specimen model indicate layers of different material properties.

The numerical experimental model that simulated the indentation tests, which is a two-dimensional axial symmetric model, is shown in Figure 4. The radius and height of the specimen were 20 and 12.5 μm, respectively. The minimum elemental size was 0.025 μm × 0.014 μm. The number of nodes and elements were 4657 and 4464, respectively. As the irradiated specimens exhibited displacement damage distribution, the irradiated area was divided into four layers. The thickness of the 1st, 2nd, 3rd, and 4th layers were 0.63, 0.56, 0.56, and 0.56 μm, respectively. The constants of the unirradiated area were defined based on the tensile test results, and three constants of each layer were identified using inverse analyses.

**Figure 5:**
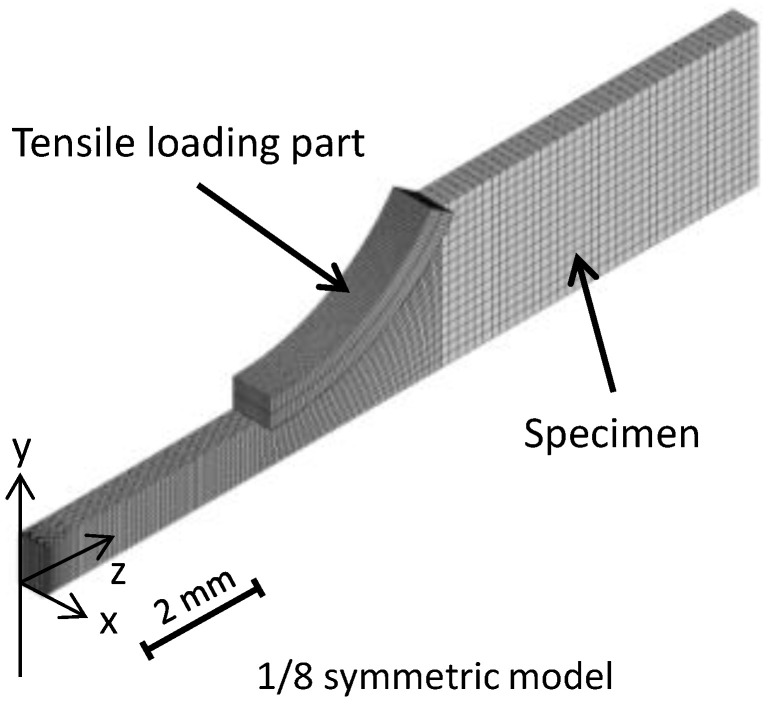
Numerical experimental model simulating tensile tests.

The elastic modulus and Poisson’s ratio of irradiated and unirradiated areas were set to 193 GPa and 0.3, respectively [53,54]. As the indenter tip was not perfectly spherical, the shape of the indenter in the numerical experimental model was corrected so that the results of the unirradiated area matched the experimental result obtained using the true stress and true strain curves derived from the tensile test result of the unirradiated specimen (Figure 5). When the experimental result was approximated using the Swift equation, the yield stress, work hardening coefficient, and work hardening exponent were 250 MPa, 1323 MPa, and 0.556, respectively. The indenter was modeled as a rigid body.

Figure 5 shows the numerical experimental model developed based on the specimen used in the tensile test because the total elongation depends on the cross-sectional shape of the specimen and the ratio of the cross-sectional area to the gauge length. The dimensions of the cross section of the center and parallel parts were 1.524 mm × 0.89 mm and 7.62 mm, respectively. Figure 5 shows the numerical experimental model used for simulating tensile tests—a three dimensional one-eighth symmetric model. To evaluate the fracture strain, the mesh density was determined to be sufficient to reproduce the deformation behavior, and the minimum size of the element was 0.014 mm × 0.027 mm × 0.048 mm. The numbers of nodes and elements were 62,605 and 54,504, respectively. A tensile loading part was modeled near the shoulder of the specimen. The shape of the surface in contact with the specimen was the same as that of the specimen. The specimen was elongated by moving the tensile loading part in the tensile direction. The tensile loading part was modeled as a rigid body.

### 3.3. Results

#### 3.3.1. Indentation Tests

**Figure 6:**
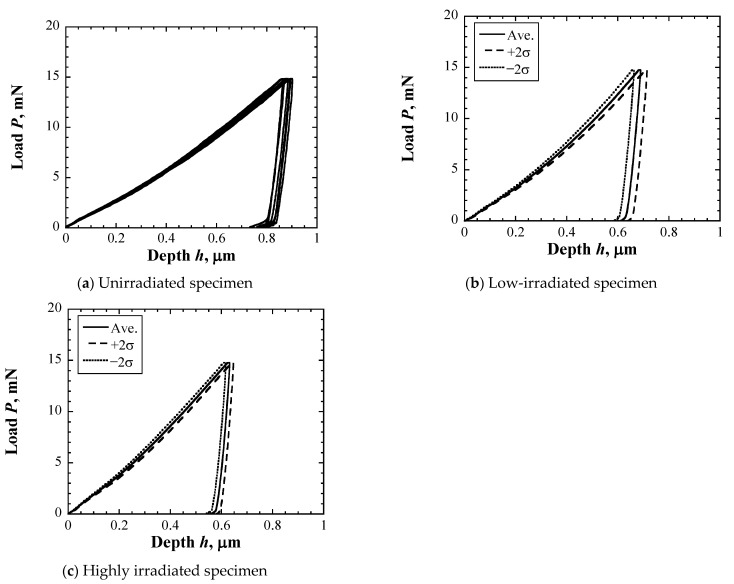
*L*–*D* curves of unirradiated and irradiated specimens.

Figure 6 shows the *L*–*D* curves of unirradiated and irradiated specimens. As the indent size was sufficiently smaller than the grain size, scatters in the measurement results include measurement errors and scatters resulting from the mechanical properties of small regions depending on the material structure. The maximum depth in the irradiated area was shallower than that in the unirradiated area, which decreased with increasing displacement damage intensity.

#### 3.3.2. Inverse Analyses Using the Kalman Filter

**Figure 7:**
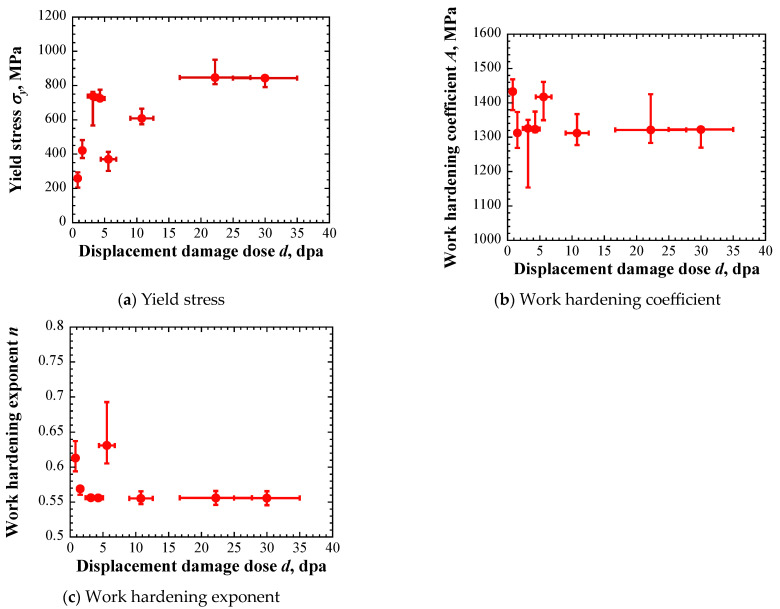
Identified constants in the material constitutive equation.

The constants for each layer in the irradiated area were determined via inverse analyses using the Kalman filter. The experimental results show scatters. The averaged *L*–*D* curves with a depth of ±2*σ* were used. Figure 7 shows the identified constants in the material constitutive equation. The red dots denote the results obtained from the averaged indentation *L*–*D* curves. The scatters in the vertical direction denote the results obtained from the curves with a scatter of ±2*σ*. The horizontal axis denotes the displacement damage intensity in each layer of two irradiated specimens (Figure 4). The identified yield stress increased with increasing displacement damage intensity, with the same trend as the experimental results. However, the identified yield stresses for the first and second layers of highly irradiated specimens were smaller than the reported experimental results, and the reason for such a result is under consideration. The identified work hardening coefficient and work hardening exponent were 1100–1500 MPa and 0.5–0.7. The two constants of the layer near the surface of low- and highly irradiated specimens were large. These results are easily affected by the specimen surface condition and contact condition. It has also been reported that the evaluation accuracy decreases when the indentation depth is shallow [21]. For these reasons, it is assumed that the results differ from other results.

#### 3.3.3. Numerical Experiments Simulating the Tensile Test

**Figure 8:**
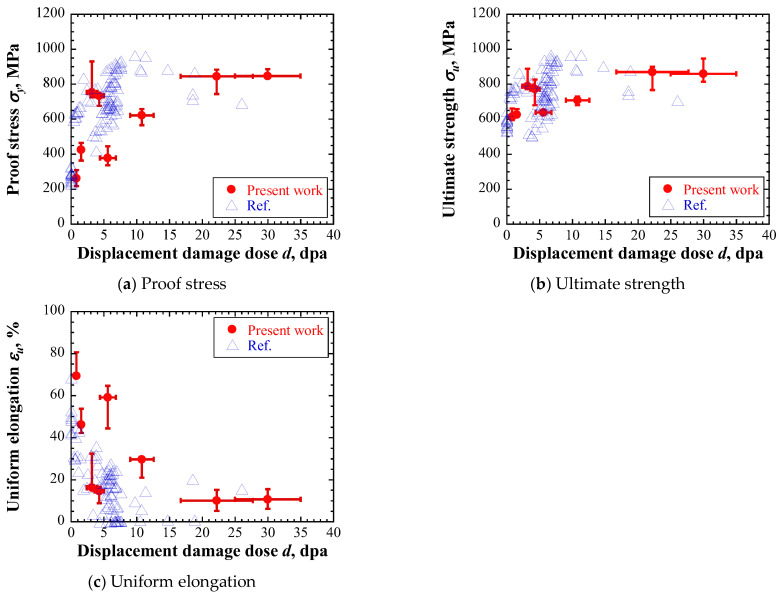
Evaluated mechanical properties based on the nominal stress and nominal strain curves as follows: (**a**) proof stress, (**b**) ultimate strength, and (**c**) uniform elongation. Blue triangles denote the reported experimental results of irradiated materials [35,36,37,38,39,40,45,46].

Numerical experiments simulating the tensile test were conducted based on the constants identified for four layers of low- and highly irradiated specimens, and then the nominal stress and nominal strain curves were obtained. The proof stress, ultimate strength and uniform elongation were derived from these curves as shown in Figure 8. Blue triangles denote the reported experimental results of the irradiated materials [35,36,37,38,39,40,45,46]. As the mechanical properties of the irradiated materials also depended on the temperature during the tensile test [55], only the experimental results at room temperature were plotted. The evaluated proof stress and ultimate strengths increased with increasing displacement damage intensity for each specimen, similar to the experimental results. However, the evaluated proof stress of the first and second layers of low-irradiated specimens and the ultimate strength of the second layer of the highly irradiated specimen were smaller than previously reported experimental results. The evaluated uniform elongations decreased with increasing displacement damage intensities of each specimen, similar to the experimental results. However, the evaluated uniform elongations for the first and second layers of highly irradiated specimens were larger than the experimental results.

#### 3.3.4. Total Elongation

(1) Method I

**Figure 9:**
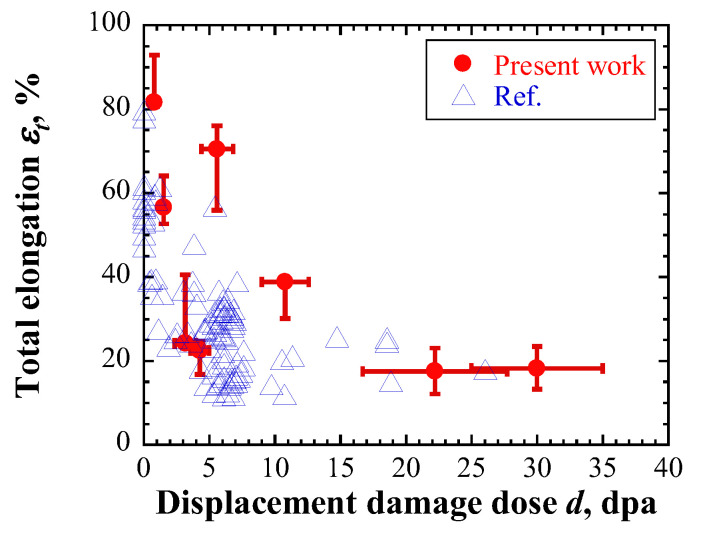
Evaluated total elongation. Blue triangles denote the reported experimental results of irradiated materials [35,36,37,38,39,40,45,46].

Figure 9 shows the total elongation calculated by substituting the evaluated uniform elongation shown in Figure 8c into Equation (3). The blue triangles indicate reported experimental results of irradiated materials [40,41,42,43,44,50,51]. The calculated total elongations decreased with increasing displacement damage intensity of each specimen, similar to the reported experimental results. However, the evaluated total elongation for the first and second layers of highly irradiated specimens was larger than the experimental result. As the total elongation was calculated based on the uniform elongation, the evaluated uniform elongation was similar to that of the experimental results.

(2) Method II

**Figure 10:**
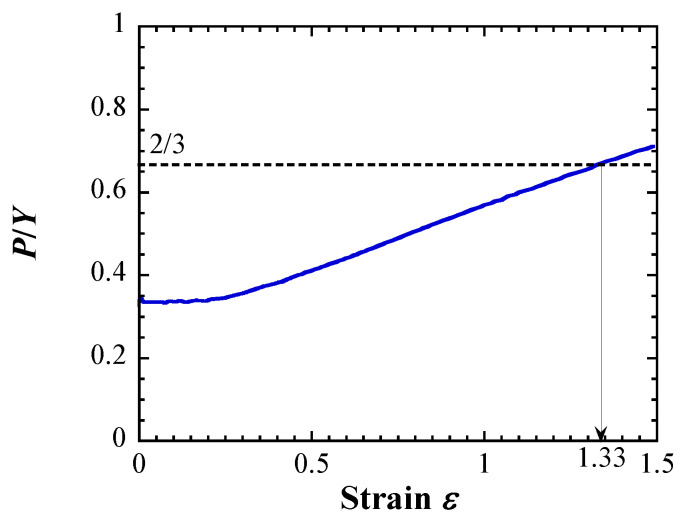
Relationship obtained from the numerical experiment using the constants identified for the 4th layer of the low-irradiated specimen with a maximum displacement damage intensity of 5 dpa.

**Figure 11:**
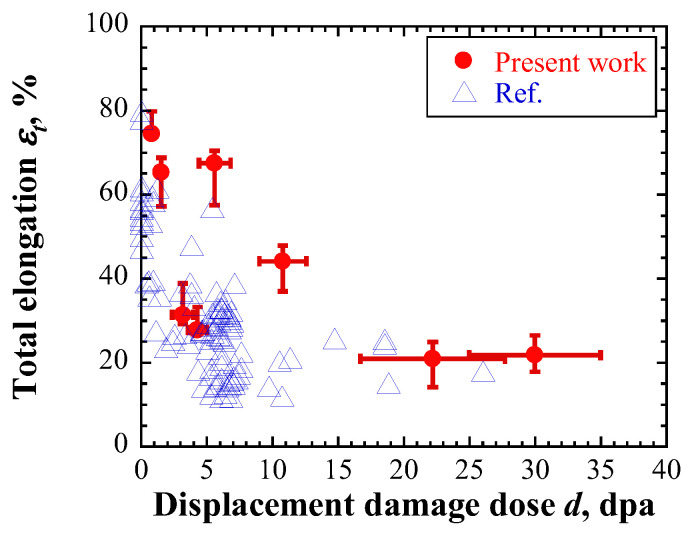
Evaluated total elongation. Blue triangles denote the reported experimental results of irradiated materials [35,36,37,38,39,40,45,46].

Figure 10 shows the relationship between *P*/*Y* and the strain obtained from the numerical experiment using the constants identified for the fourth layer of the low-irradiated specimen with a maximum displacement damage intensity of 5 dpa. *P*/*Y* was almost constant until the strain of ~0.2 and then gradually increased. When the *P*/*Y* was 2/3, the strain *ε*_0_ was 1.33. When this value was substituted into Equation (4), the calculated fracture strain *ε_f_* was 1.08. In the numerical experiment, the nominal strain was 27.8% when the strain at the center of specimen model was 1.08; this value corresponded to the total elongation. Similar evaluations of the total elongation were conducted for each layer of low- and highly irradiated specimens. Figure 11 shows the evaluated total elongations, which decreased with increasing displacement damage intensities of each specimen, similar to the experimental results of irradiated materials [35,36,37,38,39,40,45,46]. However, the evaluated total elongations for the first and second layers of highly irradiated specimens were larger than the experimental results. These findings were similar to those determined using Equation (3).

## 4. Discussions

**Figure 12:**
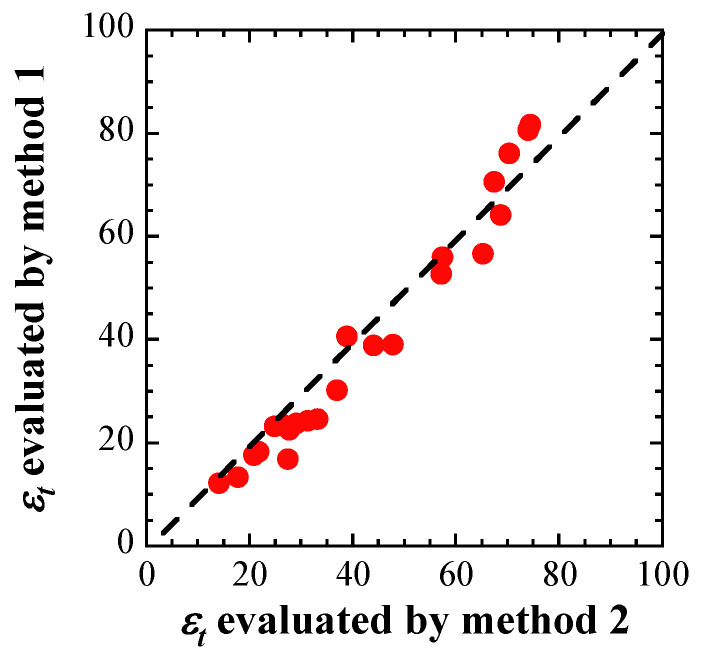
Comparison of total elongation evaluated by two kinds of procedures.

Figure 12 compares the results evaluated using Methods I and II. The total elongations evaluated using Methods I and II were almost the same, but the results evaluated using Method I were slightly smaller than those evaluated using Method II. Equation (4) used in Method II is based on the tensile test results of the ductile materials. Ductile dimples and cleavage fracture surfaces were formed on the fracture surface of irradiated materials with a displacement damage intensity of 6.8 dpa after tensile tests [46]. Equation (3) used in Method I is based on the tensile test results of irradiated materials. As the brittle characteristics of the irradiated materials also affect the tensile test results, the total elongation evaluated by Method I was deemed smaller.

## 5. Conclusions

We evaluated the ductility properties by combining numerical experiments and indentation tests, and proposed two methods to evaluate total elongation based on the results. We applied these methods to ion-irradiated materials and obtained the following results:

(1) The increasing trends in yield strength and tensile strength, as well as the decreasing trends in uniform elongation with increasing ion doses, replicate the reported experimental results of irradiated materials.
(2) The total elongation evaluated by the two methods was almost identical, which also replicates the reported experimental results of irradiated materials.

These results confirm the validity of these methods for evaluating the ductility properties of irradiated materials.

## Data Availability

The original contributions presented in the study are included in the article, further inquiries can be directed to the corresponding author.
